# An HIV-1 Envelope Glycoprotein Trimer with an Embedded IL-21 Domain Activates Human B Cells

**DOI:** 10.1371/journal.pone.0067309

**Published:** 2013-06-24

**Authors:** Gözde Isik, Nancy P. Y. Chung, Thijs van Montfort, Sergey Menis, Katie Matthews, William R. Schief, John P. Moore, Rogier W. Sanders

**Affiliations:** 1 Laboratory of Experimental Virology, Department of Medical Microbiology Center for Infection and Immunity Amsterdam, Academic Medical Center, University of Amsterdam, Amsterdam, The Netherlands; 2 Department of Microbiology and Immunology, Weill Medical College of Cornell University, New York, New York, United States of America; 3 Department of Biochemistry, University of Washington, Seattle, Washington, United States of America; 4 IAVI Neutralizing Antibody Center and Department of Immunology and Microbial Sciences, The Scripps Research Institute, San Diego, California, United States of America; 5 Department of Immunology and Microbial Science, The Scripps Research Institute, San Diego, California, United States of America; 6 IAVI Neutralizing Antibody Center, The Scripps Research Institute, San Diego, California, United States of America; 7 Scripps Center for HIV/AIDS Vaccine Immunology and Immunogen Discovery, The Scripps Research Institute, San Diego, California, United States of America; Shanghai Medical College, Fudan University, China

## Abstract

Broadly neutralizing antibodies (bNAbs) that target the HIV-1 envelope glycoproteins (Env) can prevent virus acquisition, but several Env properties limit its ability to induce an antibody response that is of sufficient quantity and quality. The immunogenicity of Env can be increased by fusion to co-stimulatory molecules and here we describe novel soluble Env trimers with embedded interleukin-4 (IL-4) or interleukin-21 (IL-21) domains, designed to activate B cells that recognize Env. In particular, the chimeric Env_IL-21_ molecule activated B cells efficiently and induced the differentiation of antibody secreting plasmablast-like cells. We studied whether we could increase the activity of the embedded IL-21 by designing a chimeric IL-21/IL-4 (ChimIL-21/4) molecule and by introducing amino acid substitutions in the receptor binding domain of IL-21 that were predicted to enhance its binding. In addition, we incorporated IL-21 into a cleavable Env trimer and found that insertion of IL-21 did not impair Env cleavage, while Env cleavage did not impair IL-21 activity. These studies should guide the further design of chimeric proteins and Env_IL-21_ may prove useful in improving antibody responses against HIV-1.

## Introduction

Ideally, an HIV-1 vaccine should activate the innate, humoral and cellular arms of the immune system and different strategies are pursued to do so. A vaccine designed to induce both B and T cell responses by combining an HIV-1 protein expressing poxvirus prime with a recombinant envelope glycoprotein (Env) boost showed 31% efficacy without inducing any bNAbs [1], [2]. The induction of broadly neutralizing antibodies (bNAbs) by Env subunit vaccines remains one of the top priorities of HIV-1 vaccine research.

Non-human primates can be protected from SHIV infection by passive immunization of bNAbs [3]–[9], but to date such bNAbs have not been induced by any vaccine. The only relevant viral protein for the induction of bNAbs is the Env spike on the surface of the virus particle. However, a number of structural properties of Env limit the induction of bNAbs. First, conserved protein bNAb targets are shielded by Env domains that are highly variable in sequence between different HIV-1 isolates [10]–[15]. Although a number of glycan-dependent bNAbs have been identified [16]–[19], the majority of Env protein domains are protected from Ab recognition by Env’s “glycan shield” [20]–[22]. Furthermore, nonfunctional Env forms on the surface of HIV-1 particles, infected cells or monomeric gp120 shed from Env trimers expose immunodominant decoy epitopes that may divert the humoral response from bNAb epitopes on functional Env trimers [23]–[26]. Although the effect on immunogenicity is not resolved, processing of the Env glycoprotein precursor gp160 into gp120 and gp41 can affect the exposure of epitopes on Env. bNAbs interact more efficiently with cleaved Env, whereas non-neutralizing Abs react more strongly with uncleaved Env [27]–[31].

These properties influence the specificity of the Ab response, i.e. they favor the induction of non-neutralizing Abs over bNAbs. There is also evidence that Env directly modulates the quantity and the quality of the Ab response to itself. The Ab response against Env requires multiple booster vaccinations and wanes quickly with a half-life of 30–60 days [32], [33]. One explanation is that *N*-linked oligomannose glycans on Env actively suppress immune cell functioning [34]–[37]. Indeed, vaccination studies in mice showed that de-mannosylated gp120 was more immunogenic than unmodified gp120 [38]. Taken together, a variety of Env properties may reduce its immunogenicity.

The abilities of Env to circumvent the host immune responses oblige vaccinologists to search for unconventional approaches to improve its immunogenicity. The co-delivery of immunogens with adjuvants is a frequently used strategy to improve the immunogenicity of a vaccine. Moreover, the immunogenicity of vaccines can also be increased by co-deliverying genes encoding molecular adjuvants, including but not limited to cytokines and chemokines such as IL-12, IL-28, GM-CSF, and IL-15 [39], [40]. The addition of costimulatory molecules also provides an opportunity to skew the immune response in a desirable direction.

We are pursuing a vaccine strategy in which we covalently link the antigen (i.e. Env) with the molecular adjuvant. We have previously described soluble gp140 Env trimers with an embedded Granulocyte Macrophage Colony Stimulating Factor (GM-CSF) [41], [42] a survival, proliferation and differentiation factor for several hematopoietic precursor cell populations [43]–[45]. The chimeric Env_GM-CSF_ protein was created by substituting the variable loops 1 and 2 (V1V2) of Env with an almost complete GM-CSF sequence. Env trimers with embedded GM-CSF induced enhanced Env-specific antibody and T helper responses in mice [42]. We have also shown that targeting Env trimers to B cells via fusion to APRIL, which is an important co-stimulatory molecule for humoral immunity that drives antibody class-switching toward IgG and IgA and plasma cell survival [46]–[48], enhances Env-specific antibody responses [49].

We hypothesized that the common gamma chain family members interleukin-4 (IL-4) and in particular interleukin-21 (IL-21), which have a similar four-helix structure as GM-CSF [50], might be useful to aid the immunogenicity of Env. IL-4 and IL-21 are pleiotropic cytokines acting on both innate and adaptive immune responses. IL-21 augments antibody production, and class switching by B cells [51], [52], and drives the differentiation of B cells to antibody secreting plasma cells [51], [53]. IL-21 is produced by multiple T helper populations such as activated CD4^+^ T cells, natural killer T (NKT), and in particular T-follicular helper cells [54]–[56]. IL-21 autocrinely activates follicular helper cells in germinal centers [57], [58] where mature B cells rapidly proliferate, differentiate, and go through somatic hypermutation and class-switching processes during a normal immune response to an infection [59]. Finally, IL-21 can activate CD8^+^ T cells to become killer cells by increasing their granzyme B and perforin expression [60], [61]. The immunomodulatory roles of IL-21 have raised considerable interest in its therapeutic use, and it has been evaluated in a number of clinical trials against, for example, metastatic melanoma and renal cancer [60], [62]. So far, these clinical trials have provided IL-21 with a good clinical track record in terms of safety.

Previous studies have shown that the function of recombinant IL-21 can be improved. For example, IL-21 signaling can be enhanced by 10-fold by replacing a structurally unstable region of IL-21 around helix C and the CD loop with the homologous and structurally more stable region from IL-4, probably preforming IL-21 in the receptor bound state [63]. Furthermore, Kang *et al.* predicted IL-21 residues important for interaction with the α and the γC chains of the IL21 receptor based on homology modeling and alignment with related cytokines such as IL-2 and IL-4 and investigated these residues by mutagenesis [64]. Three mutants were identified (D18A, S113A, and K117A) that have a slightly increased γC binding capacity, most likely due to a slower dissociation rate compared to wild type hIL-21. Other mutants had increased affinity for the IL-21R α chain (R11A, E100A, Q116A and L123A).

Here, we investigated whether trimeric HIV-1 Env proteins with IL-4 or IL-21 incorporated into the V1V2 domain could activate human B cells. In addition, we evaluated a number of IL-21 variants. We present evidence that a number of chimeric Env_IL-21_ constructs potently activate B cells and induce immunoglobulin secretion from these cells. These chimeric proteins might be useful as vaccines aimed at inducing humoral immunity against HIV-1.

## Results

### Design of HIV-1 Env trimers with an Embedded IL-4 or IL-21 Domain

**Figure 1:**
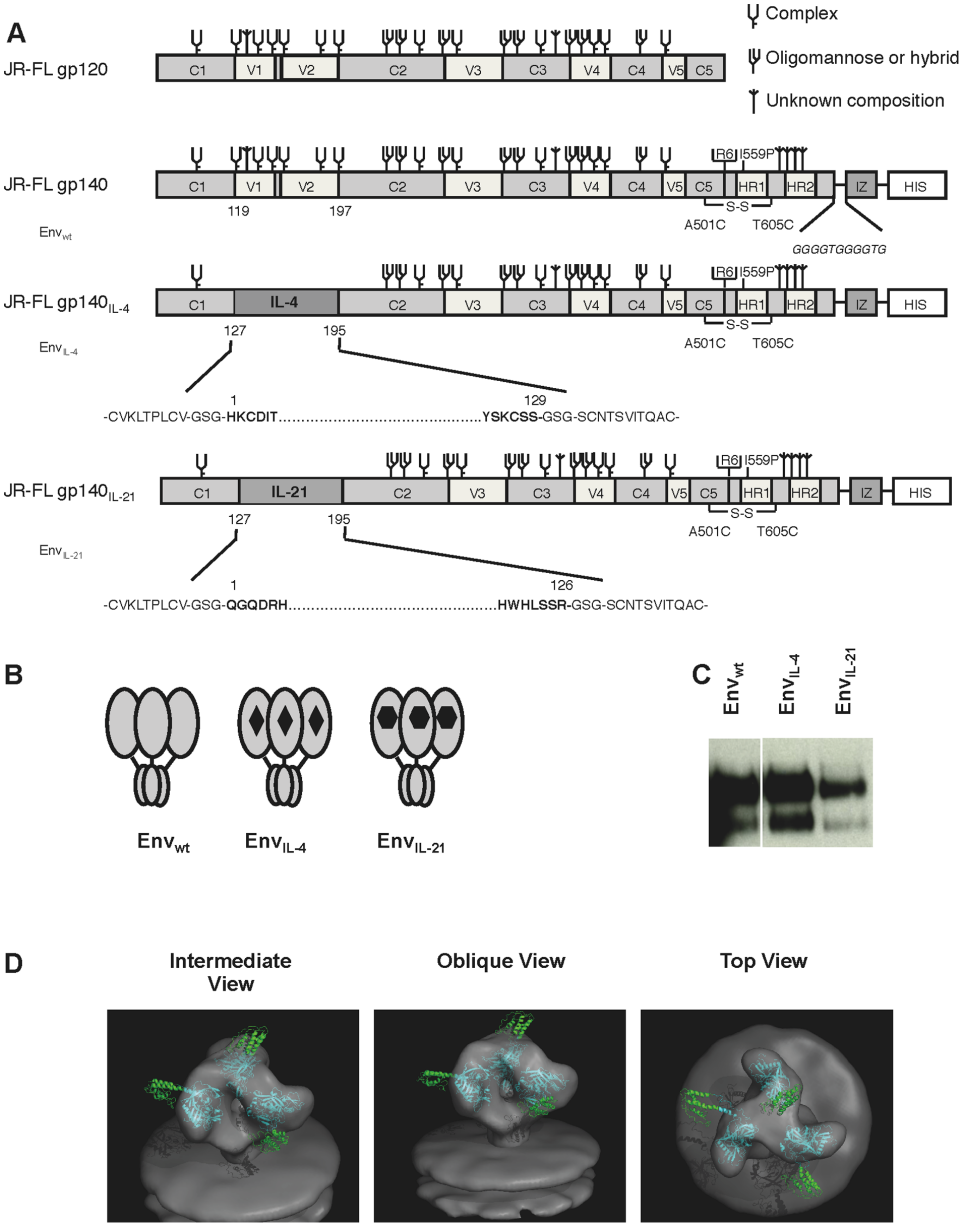
Schematics and expression of the Env_wt_, Env_IL-4_ and Env_IL-21_ molecules. Linear (A) and cartoon (B) representation of the original Env gp140 and chimeric Env_IL-4_ and Env_IL-21_. The clade B JRFL gp140 protein (amino acids 31 to 681) contains several modifications for stabilization that have been previously described (see Materials and Methods). Codon optimized sequences encoding human IL-4 (129 amino acids) and IL-21 (126 amino acids) were inserted to the V1V2 domain of gp140. Env sub-domains are indicated: 5 conserved domains (C1–C5); 5 variable domains (V1–V5); heptad repeats 1 and 2 (HR1, HR2); the trimerization domain (IZ) and the histidine tag, comprised of 8 histidine amino acids (HIS). The glycan assignments in Env are based on previous studies using gp120 [85]–[87]. (C) Chimeric Env_IL-4_ and Env_IL-21_ proteins expressed transiently in 293T cells were analyzed in reducing SDS-PAGE analysis followed by western blot. (D) Models of Env_IL-21_ trimers. hIL-21 (green) is shown in up, side or down orientations attached to gp120 (cyan). The models were generated using Chimera [82], RosettaDesign [84] and RosettaRemodel as described in the materials and methods section, and rendered using Pymol [88].

With the aim of targeting HIV-1 Env to B cells and simultaneously activating these cells, we designed chimeric Env constructs by replacing the V1V2 domain of gp140 with the nearly complete sequence of human interleukin 4 (IL-4) or interleukin 21 (IL-21). The uncleaved gp140, which is fused to a C-terminal trimerization domain, is based on the JR-FL strain and is described in more detail elsewhere [11], [27], [65]–[67]. The removal of the V1V2 domain is also described elsewhere [42], [68]. Briefly, 129 amino acids of IL-4 (residues 1 to 129) or 126 amino acids of IL-21 (residues 1 to 126) were inserted after the second cysteine bridge in the V1V2 stem between amino acids 127 and 195 (Fig. 1A & 1B). Three amino acid long linkers were added to the N and C-terminus of IL-4 and IL-21 to ensure flexibility at the junctions of the cytokine domains and Env.

We transiently transfected 293T cells with plasmids encoding gp140 (Env_wt_), gp140_IL-4_ (Env_IL-4_) and gp140_IL-21_ (Env_IL-21_). The expression of Env_IL-4_ was comparable to Env_wt_ whereas that of Env_IL-21_ was decreased. The Env_IL-4_ and Env_IL-21_ proteins were approximately equal in size compared to Env_wt_ (Fig. 1C). Some gp120 was also visible, derived from cleaved Env. As reported previously, JRFL gp140 with a C-terminal trimerization domain is not cleaved efficiently between gp120 and gp41, resulting in uncleaved gp140 as the predominant species although some cleavage does occur yielding gp120 [42], [68], [69].

We generated a structural model of the Env_IL-21_ trimer based on the atomic structures of gp120 [70], a gp120 trimer model [71], the structure of IL-21 [63], and additional information we obtained from the antigenicity and functional data (see below). The hIL-21 sequence was grafted onto the V1V2 stem. An Env_hIL-21_ trimer and IL-21 with different orientations in all three protomers is shown in Fig. 1D. All the models are compatible with VRC01, b12 and CD4 binding. Due to the flexibility of the linkers, IL-21 can be modeled on the gp120 core in multiple orientations and the number of conformational possibilities is quite large. The construct is likely to sample all possible orientations.

### Chimeric Env_IL-4_ and Env_IL-21_ Interact with Conformational Antibodies

**Figure 2:**
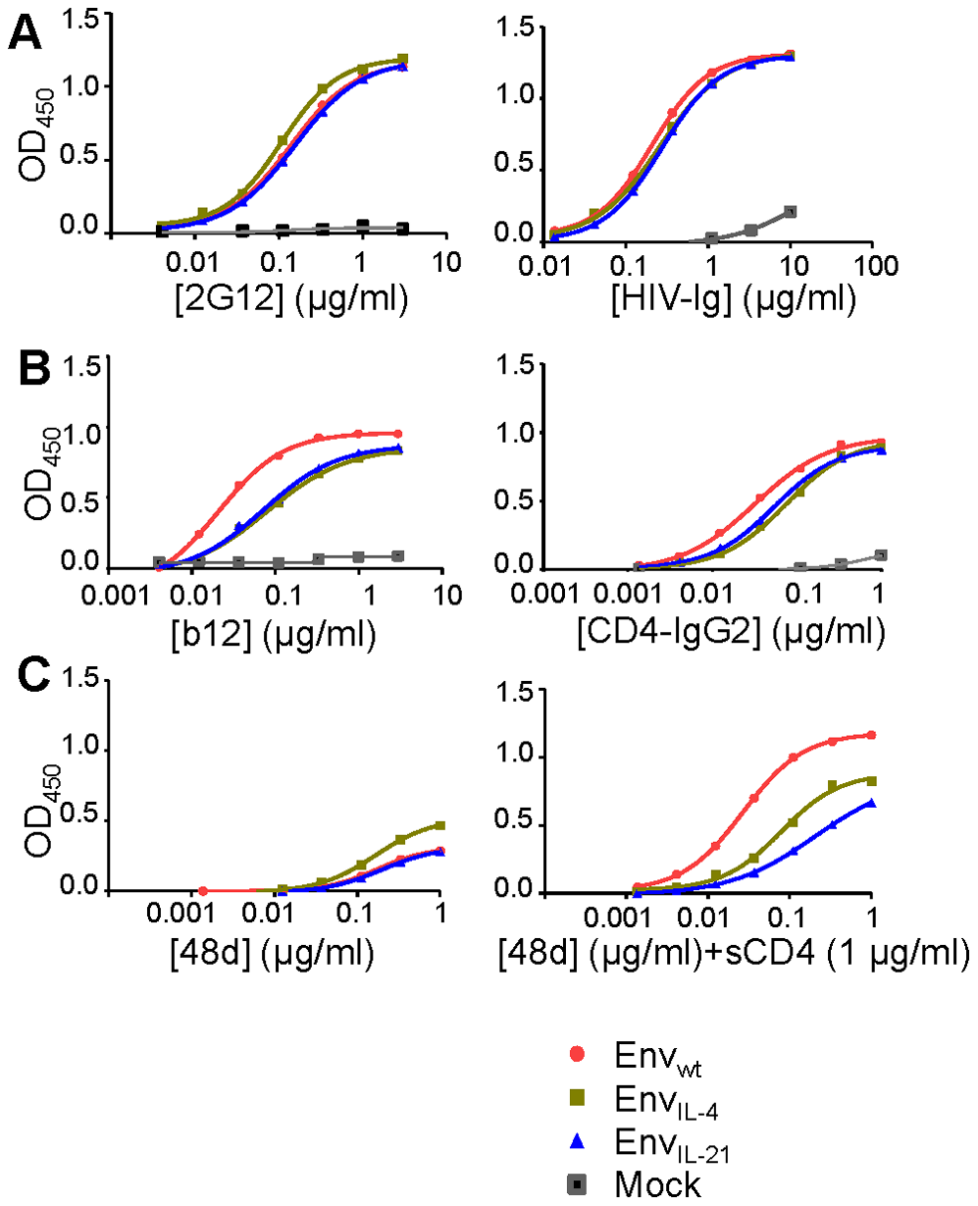
Antigenic characterization of Env_IL-4_ and Env_IL-21_ molecules. ELISA reactivity of Env_IL-4_ and Env_IL-21_ with 2G12 and HIV-Ig (A); b12 and CD4-IgG2 (B); and 48d (CD4i) in the absence and presence of sCD4 at 1 µg/ml (C). All ELISA results are representative for at least three independent experiments using proteins derived from three independent transfections.

We evaluated the antigenic structure and folding of Env_IL-4_ and Env_IL-21_ compared to Env_wt_ by using a trimer ELISA. The oligomannose *N-*glycan binding 2G12 bNAb bound to Env_IL-4_ and Env_IL-21_ as efficiently as to Env_wt_ while binding of HIVIg (pooled polyclonal Ig from HIV-positive patient sera) was slightly reduced, probably due to the lack of the V1V2 domain (Fig. 2A). The binding of the CD4 binding site (CD4bs) b12 bNAb and the receptor mimic CD4-IgG2 was slightly reduced for both Env_IL-4_ and Env_IL-21_, consistent with what we have observed previously for Env_GM-CSF_ and other Env_GM-CSF_ variants (Fig. 2B) [41], [42]. The CD4-induced (CD4i) MAb 48d did not bind efficiently to any of the three constructs in the absence of CD4. In the presence of CD4, 48d bound efficiently to Env_wt_, but its binding to Env_IL-4_ and Env_IL-21_ was limited, consistent with our previous findings that replacement of the V1V2 with a cytokine domain partially traps Env in the unliganded-state and blocks CD4-induced conformational changes (Fig. 2C) [41].

### Chimeric Env_IL-21_ Drives the Differentiation of Humans B Cells into Immunoglobulin Secreting Plasmablasts

**Figure 3:**
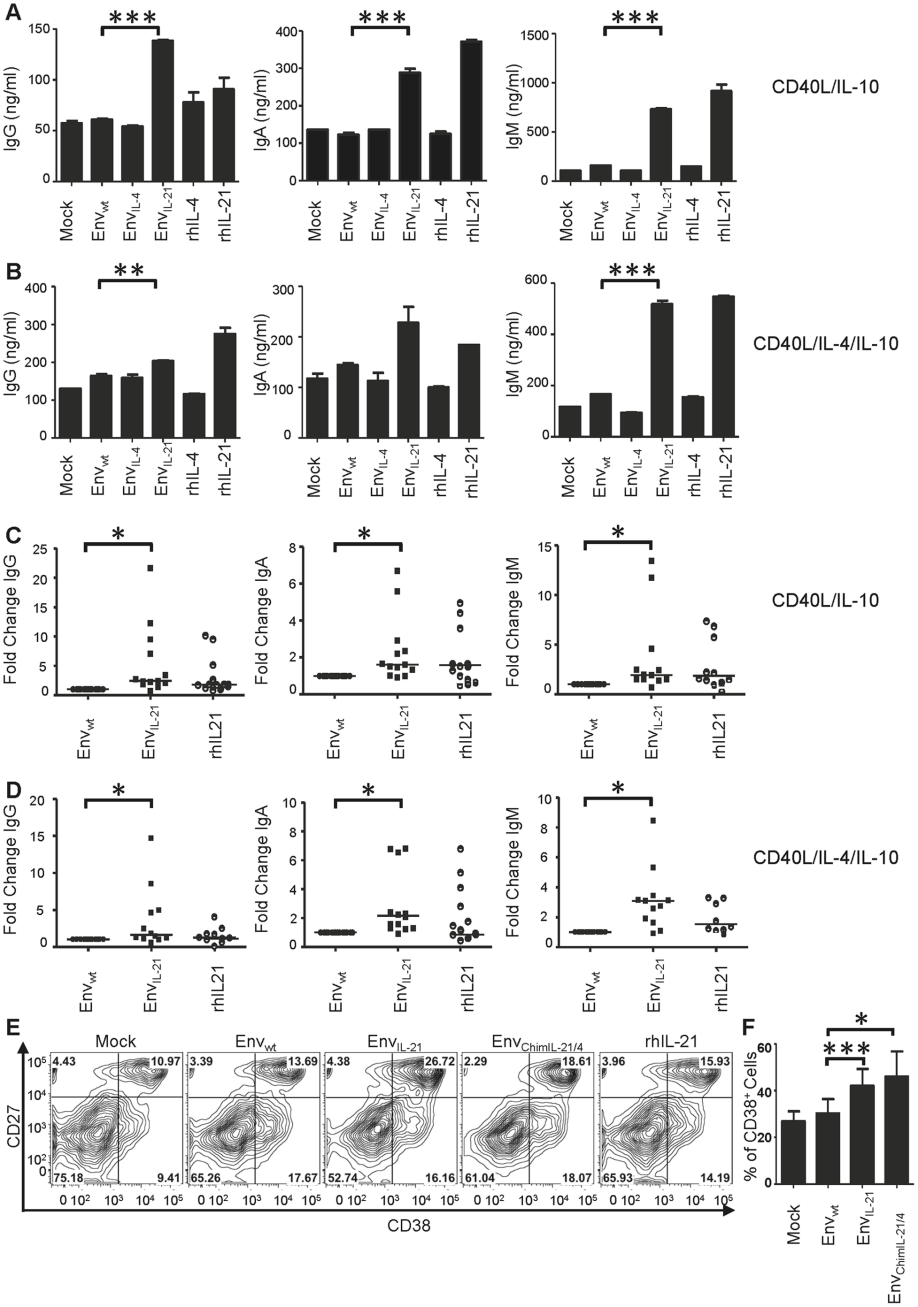
Immunoglobulin production by B cells stimulated with Env_wt_, Env_IL-4_ and Env_IL-21_ molecules and controls. IgG, IgA and IgM levels secreted by the B cells from human PBMCs cultured with (A) CD40L/IL-10 and (B) CD40L/IL-4/IL-10. Data are representative of three independent experiments showing similar results. Immunoglobulin secretion by B cells from different donors cultured with Env_wt_ and Env_IL-21_ molecules in (C) CD40L/IL-10 and (D) CD40L/IL-4/IL-10 milieu. Culture supernatant from mock transfected 293T cells was used as a negative control and the values were deducted from the test values. Data represent the fold change values compared to Env_wt_ from at least 12 donors and each donor sample was tested in duplicate. (E) The expression of cell surface markers CD38 and CD27 on B cells cultured with Env_wt_, Env_IL-21,_ Env_ChimIL-21/4_ supernatants and controls in CD40L/IL-10 milieu. Data are representative of three experiments using B cells from three different donors. (F) The expression of CD38 cell surface marker treated with different Env_IL-21_ constructs and controls in CD40L/IL-10 milieu. Data are representative of six experiments using B cells from six different donors.

The functionalities of the IL-4 and IL-21 inserted into Env were tested by performing experiments with purified human B cells. The B cells were isolated from human PBMC and cultured in the presence of supernatants from 293T cells expressing Env_wt_, Env_IL-4_, Env_IL-21_ and untransfected cells (Mock) or alternatively with pure recombinant IL-4 (rhIL-4) and IL-21 (rhIL-21), in a background milieu containing CD40L and IL-10 (Fig. 3A) or CD40L, IL-10 and IL-4, to support B cell activation (Fig. 3B). The Env protein levels for different constructs were normalized based on an anti-Env ELISA using the 2G12 MAb. After 14 days of culture, total IgG, IgA and IgM levels in the supernatants were determined by an immunoglobulin ELISA. The wild type Env protein (Env_wt_) induced low levels of IgG, IgA and IgM from B cells, consistent with previous reports demonstrating that Env can activate B cells through binding to lectin receptors [72]. Env_IL-21_ significantly and consistently augmented the total IgG, IgA and IgM levels in both CD40L/IL-10 and CD40L/IL-4/IL-10 supplemented environments, although the level of enhancement was highly dependent on the donor (Fig. 3C & 3D). In some donors, the fold change values of Env_IL-21_ compared to Env_wt_ were as high as 21, 6.5 and 13 for IgG, IgA, and IgM, respectively. Compared to unmodified Env (Env_wt_), we did not observe a consistent enhancement in immunoglobulin secretion from B cells treated with Env_IL-4_ (Fig. 3A & 3B).

We next evaluated the expression of surface markers on B cells cultured with Env_wt_, Env_IL-21_ supernatants and controls, again in a milieu that contained CD40L and IL-10. Unmodified Env induced the up-regulation of the B cell activation marker CD38 and the memory B cell marker CD27 (20% double positive cells), indicative of a plasmablast phenotype, consistent with previous reports [72]. Env_IL-21_ further enhanced the numbers of CD38^+^CD27^+^ plasmablast-like cells (26%) similar to the level reached with rhIL-21 (26%) (Fig. 3E). Env_IL-21_ significantly increased the CD38 expression on B cells (42%), compared to mock (26%) and Env_wt_ (30%) treated cells (Fig. 3F). Thus, Env_IL-21_ facilitates the induction of Ig-secreting plasmablast-like cells, showing that the inserted IL-21 domain is functional.

We have investigated a number of strategies to improve the IL21 function of Env_IL-21_ based on previous studies but did not succeed in doing so (See Results S1 & Figures S1, S2, S3, S4).

### Env Cleavage does not Interfere with the Bioactivity of Env_IL-21_

**Figure 4:**
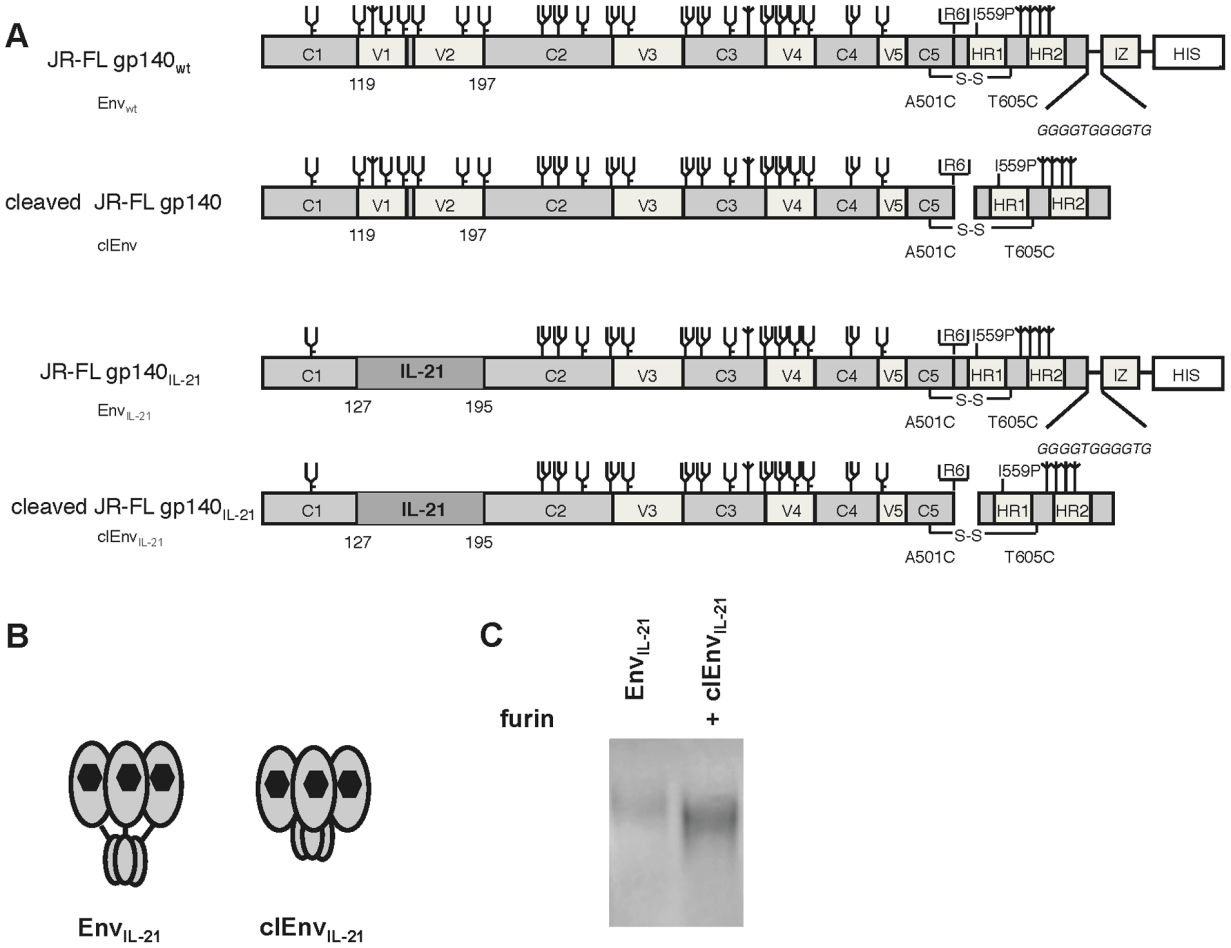
Schematic and expression of the cleavable Env_IL-21_. Linear (A) and cartoon (B) representation of the Env_IL-21_ and clEnv_IL-21_ proteins. Cleaved proteins were created by introducing a stop codon in front of the isoleucine zipper (IZ) trimerization domain in the Env_IL-21_. (C) SDS-PAGE analysis of chimeric uncleaved and cleaved Env_IL-21_ constructs.

Normal Env function requires cleavage of the Env precursor gp160 into the gp120 and gp41 subunits, and cleavage is also required for an optimal antigenic structure. Although cleaved soluble trimers might be better mimics of Env on the virions, their instability makes them difficult to study. Hence, several protein-engineering strategies have been employed to stabilize soluble Env trimers that include mutation of the cleavage site and introduction of heterologous trimerization domains. In our gp140 constructs, the cleavage site is intact and an intermolecular disulfide bond holds the gp120 and gp41 subunits together [67]. However, to increase the trimer stability, we also added a heterologous trimerization domain, and this interferes with Env cleavage [49], [66], [69]. To assess the influence of the inserted IL-21 domain on cleavage as well as the effect of cleavage on IL-21 activity, we removed this trimerization domain (isoleucine zipper (IZ)) from the Env_IL-21_ construct (Fig. 4A & 4B).

**Figure 5:**
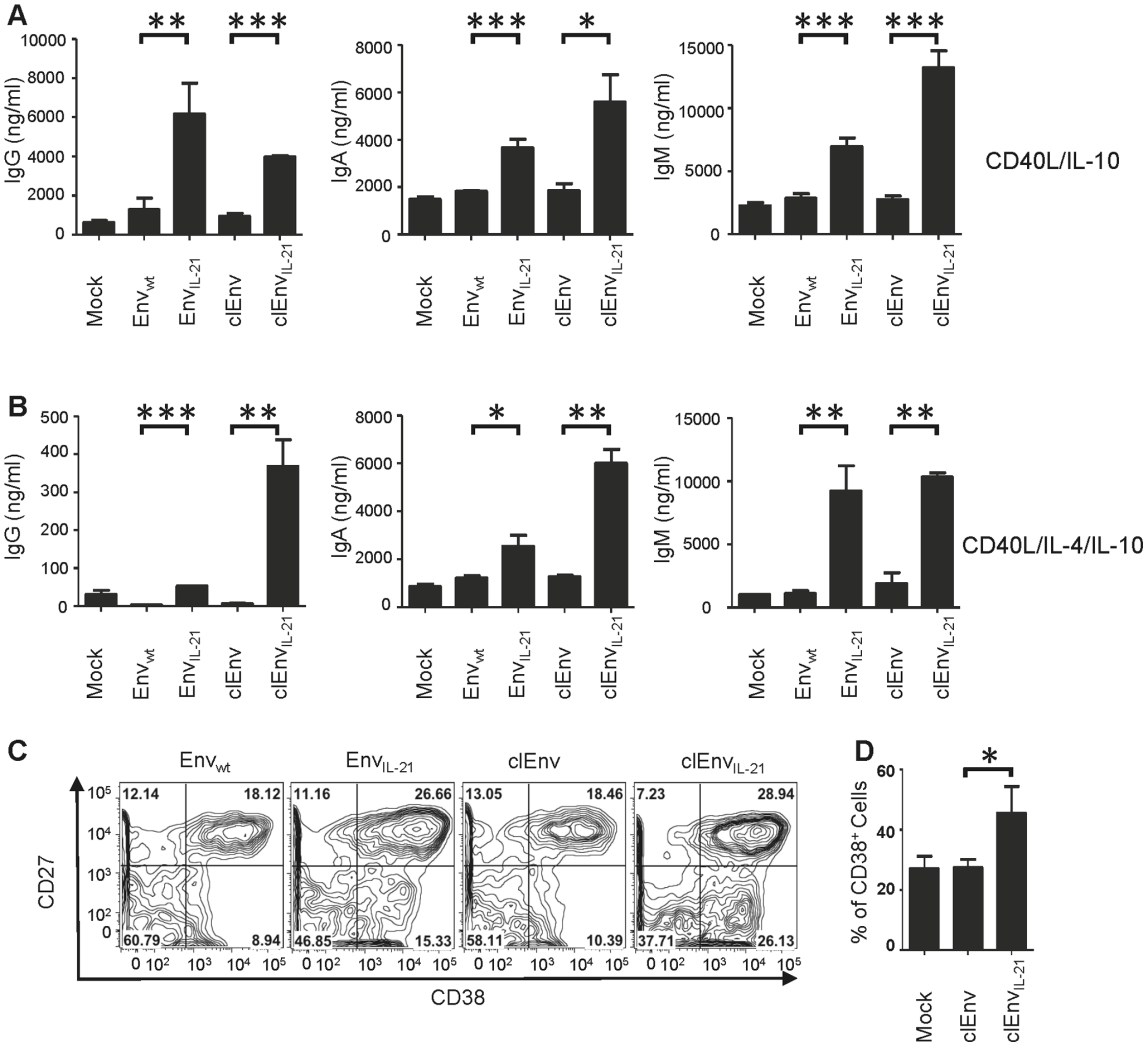
Immunoglobulin secretion from B cells cultured with Env_wt_, Env_IL-21_, clEnv and clEnv_IL-21_ molecules in the presence of (A) CD40L/IL-10 and (B) CD40L/IL-4/IL-10. Data are representative of two independent experiments using B cells from two different donor, each tested in duplicate. (C) The expression of cell surface markers CD38 and CD27 on B cells cultured with Env_wt_, Env_IL-21_, clEnv and clEnv_IL-21_supernatants in medium supplemented with CD40L/IL-10. Data are representative of three independent experiments using B cells from three donors. (D) The expression of CD38 cell surface marker treated with different cleaved Env (clEnv) and cleaved Env_IL-21_ (clEnv_IL-21_) constructs in CD40L/IL-10 milieu. Data are representative of six experiments using B cells from six donors.

We measured the cleavability of the Env_IL-21_ trimers by subjecting them to SDS-PAGE analyses under reducing conditions. To increase the cleavage of cleavable Env protein, we co-delivered a plasmid encoding the Furin protease [65]. Expression of cleavable Env_IL-21_ (clEnv_IL-21_) was higher than uncleaved Env_IL-21_, and the cleavable Env_IL-21_ (clEnv_IL-21_) migrated faster through the gel indicating that the gp41 ectodomain was indeed removed (Fig. 4C). Next, the effect of Env cleavage on the activity of IL-21 was tested on B cells. The clEnv_IL-21_ trimers were capable of augmenting the secretion of IgG (3.2±0.18 & 0.4±0.12 µg/ml), IgA (5.6±2.5 & 6±0.8 µg/ml) and IgM (13±3 & 10±0.4 µg/ml) from human B cells both in the CD40L/IL-10 & CD40L/IL-4/IL-10 containing milieus, whereas less IgG, IgA and IgM secretion was induced by cleaved Env_wt_ (clEnv) (IgG 0.9±0.3 & 6.4±2.5, IgA 1.8±0.5 & 1.3±0.08, IgM 2.7±0.5 & 1.9±1.2 µg/ml), similar to the induction by uncleaved Env_wt_ (Fig. 5A & 5B). Moreover, clEnv_IL-21_ efficiently induced the differentiation of B cells towards CD27^+^CD38^+^ plasmablast-like cells (29%) (Fig. 5C) similar to Env_IL-21_ (27%), whereas clEnv_IL-21_ (19%) and mock supernatant treated B cells differentiated to a lesser extent (10–15%, data not shown). Compared to clEnv (27%), clEnv_IL-21_ (45%) significantly increased the CD38 expression on B cells (Fig. 5D).

## Discussion

Currently, no HIV-1 Env-based subunit vaccine has been successful at inducing long-term protective humoral immunity against HIV-1. Adjuvant formulations can improve the immunogenicity of subunit vaccines in many ways, for example by activating professional immune cells and actively attracting them to the injection site. As an alternative approach to mixing antigen and adjuvant (or a co-stimulatory molecule), in which case adjuvant and antigen might be separated from each other by diffusion after injection, and hence interact with different immune cells, we have been following a strategy that entails covalently linking the antigen to the costimulatory molecule to ensure that the antigen and adjuvant interact with the same immune cells. Such construct we created was a chimeric molecule in which GM-CSF was incorporated into Env (Env_GM-CSF_); it enhanced both T and B cell responses to Env in mice [42]. Our second chimeric molecule had an APRIL moiety fused to the C-terminus of Env trimers (Env_APRIL)_; it activated human B cells, and induced better NAb responses to HIV-1 in rabbits [49], [69].

The two above studies provided the rationale for the current one. Here, we tested whether it would be possible to incorporate the B cell activating cytokine IL-21 into the Env V1V2 domain. IL-21 is important for generating and sustaining high affinity antibody responses *in vivo* and is a very potent inducer of plasma cell differentiation [52], [53], [73]–[75]. This property of IL-21 is of relevance for raising bNAbs because they usually contain a large number of somatic mutations [76]. Here, we describe a chimeric molecule that induces the differentiation of B cells into immunoglobulin secreting cells with a plasmablast-like phenotype.

When designing chimeric molecules, many restrictions, such as size, molecular weight and structure may limit the possibility of obtaining a well-folded antigen and a functional costimulatory molecule. Therefore we took our successfully engineered Env_GM-CSF_ as the template for the current study [41], [42]. The common γ chain cytokines IL-4 and IL-21 share the same four-helix bundle structure with GM-CSF; we anticipated that this would increase the likelihood of creating functional chimeric molecules [42], [50]. We could not detect the activity of Env_IL-4_ on B cells, but this may be due to our experimental system as we did not observe activity for rhIL-4 under these conditions either. In other studies, we found that Env_IL-4_ was capable of differentiating monocytes as monitored by downregulation of CD14, although it was not very potent at doing so (data not shown).

We cannot directly compare the functionality of the cytokine domains of Env_IL-21_ and Env_GM-CSF_ in an *in vitro* assay because these cytokines have distinct biological properties and target different cell types. However, we did compare Env_IL-21_ with the Env_APRIL_ molecule that we previously described [49]. Interestingly, Env_IL-21_ was considerably more potent at activating purified human B cells and inducing immunoglobulin secretion from purified human B cells *in vitro* (data not shown). We are planning immunization studies to compare the overall immunogenicity of Env_IL-21_, Env_APRIL_, Env_GM-CSF_, as well as other Env-fusion molecules *in vivo*.

The unmodified Env_IL-21_ protein activated human B cells to secrete immunoglobulins and differentiate into plasmablast-like cells, but we were not successful in increasing the activity further. We did observe that replacing a region around helix C and the CD loop of IL-21 in Env_IL-21_ by the corresponding region from IL-4 rescued the low expression of the Env_IL-21_ protein, while preserving IL-21 activity. Hence this unstable region could play a role in the low expression of Env_IL-21._ However, Env_ChimIL-21/4_ did not consistently enhance the activation of human B cells compared to Env_IL-21_, contrary to what was found in a reporter assay using *E.coli* produced recombinant IL-21 [63]. Although shortening the CD loop may reduce flexibility and increase receptor signalling, specific contacts between IL-21 and the IL-21 receptor that are needed for full functionality might be lost upon introduction of the IL-4 segment [63]. We also introduced substitutions into the IL-21 domain of Env_IL-21_ to increase the affinity of IL-21 to its receptor complex, based on previous receptor affinity studies. The selected mutations were previously shown to increase the affinity to one component of the IL-21 receptor complex (either the α- or the γ-chain), while decreasing the affinity for the other receptor chain [64]. However, none of the selected variants increased the potency of Env_IL-21._ In contrast, except for the S113A mutant, all the selected mutations abolished the activity of Env_IL-21_ on B cells, suggesting that a balanced interaction with both IL-21 receptor chains is required for optimal activity of Env_IL-21_.

In our view, an ideal Env antigen should expose all or most known bNAb epitopes. Obviously, chimeric Env_IL-21_ does not expose bNAb epitopes in the V1V2 domain such as those for PG9, PG16 and PGT145, for the simple reason that this domain was removed. The limited antigenic analyses we performed indicate that other Env epitopes in Env_IL-4_, Env_IL-21_, Env_ChimIL-21/4_ were similarly exposed as on Env_wt_, although b12 and CD4 itself bound slightly less efficiently. Furthermore, binding of MAb 48d to the CD4i epitope was reduced even in the presence of CD4. This is consistent with what we have observed previously for Env_GM-CSF_. We suggest that intrinsic properties of the V1V2 allow CD4-induced conformational changes to occur and that replacing the V1V2 with a cytokine locks Env into the unliganded state [41].

The native Env spike on the virus is cleaved between gp120 and gp41, an event needed for Env function. Hence, an ideal (soluble) mimic of the Env spike should also be cleaved. In general, non-neutralizing antibodies favor uncleaved Env over cleaved Env while the opposite is true for bNAbs [24], [28]–[31], [65]. Therefore, we investigated whether IL-21 could be incorporated into cleaved gp140 constructs and found that Env cleavage did not interfere with the functionality of IL-21. The presence of IL-21 also did not affect Env cleavage, consistent with our previous findings with the Env_GM-CSF._ Hence the V1V2 domain at the apex of the Env trimer does not influence cleavage of Env glycoprotein trimer [41].

We have generated a chimeric Env_IL-21_ protein that combines a reasonably good antigenic structure of Env with the immunostimulatory effect of IL-21. The chimeric Env_IL-21_ is not a perfect mimic of the Env spike on a virus, as exemplified by the lack of the epitopes of bNAbs such as PG9, PG16 and PGT145 [77]–[80]. Furthermore, Env_IL-21_ of course lacks the epitopes for Abs directed to the V1V2 domain. Although we are actively working on Env_IL-21_ chimeras that preserve the V1V2 domain, it remains to be seen whether the sacrifice of some possibly advantageous properties outweighs the beneficial effect of the insertion of IL-21 [81]. In conclusion, we have designed a new candidate Env immunogen, aimed at augmenting the B cell response against Env that might be worth testing in animal models.

## Methods

### Constructs

The plasmid expressing codon-optimized stabilized HIV-1 gp140 (SOSIP.R6-IZ; [11], [27], [65]–[67]) was used as the starting point for the constructs generated in this study (Fig. 1A). The Env is based on the subtype B, CCR5-using primary isolate JR-FL and is described in detail elsewhere [11], [27], [65]–[67]. Amino acid numbering is based on HXB2 gp160 according to convention. Codon-optimized DNA encoding human interleukin 4 (IL-4) and interleukin 21 (IL-21) flanked by HindIII and BmgBI restriction sites was synthesized (Mr. Gene Regensburg, Germany). The V1V2 domain of Env was exchanged with the sequences coding for IL-4 and IL-21 using the HindIII and BmgBI sites. Substitutions and insertions were generated using the QuikChange™ mutagenesis kit (Stratagene, CA, USA) and the sequence integrity of all constructs was verified by sequencing.

### Reagents

HIV-Ig was obtained through the AIDS Research and Reference Reagent Program (ARRRP), Division of AIDS, NIAID, NIH. MAb 2G12 was obtained from Hermann Katinger through the ARRRP. CD4-IgG2, sCD4 and anti-V3 gp120 MAb PA1 were gifts from Bill Olson (Progenics Pharmaceutical, Tarrytown, NY). MAb b12 was donated by Dennis Burton (The Scripps Research Institute, La Jolla, CA). MAb 48d was a gift from James Robinson (Tulane University, New Orleans, LA). Recombinant human IL-21 (rhIL-21) (used at 10 ng/ml) was obtained from PeproTech (London, UK).

### Cells Lines and Transfections

293T cells [42] were maintained in Dulbecco’s Modified Eagle’s Medium (DMEM; Invitrogen, Breda, The Netherlands) supplemented with 10% heat inactivated fetal calf serum (FCS; HyClone, Perbio, Etten-Leur, the Netherlands), MEM nonessential amino acids (0.1 mM; Invitrogen, Breda, the Netherlands) and penicillin/streptomycin (both at 100 U/ml). 293T cells were transiently transfected with plasmids expressing recombinant Env using the Nanofectamine reagent following the manufacturer's recommendation (PAA, Pasching, Austria). For cleavable Env, a plasmid encoding the furin protease was co-transfected at a 1∶2 ratio with the Env encoding plasmid [65]. Env containing supernatants were harvested 48 h after transfection and frozen in aliquots.

### SDS-PAGE and Western Blotting

SDS-polyacrylamide gel electrophoresis (SDS-PAGE) and western blotting were performed as previously described [66]. Env was detected using the MAb PA1 (0.2 µg/ml) and a 1∶5,000 diluted secondary HRP-labeled goat-anti-mouse IgG (Kirkegaard & Perry Laboratories, Maryland, USA) followed by detection using the Western Lightning ECL solution (PerkinElmer, Groningen, The Netherlands).

### Molecular Modeling

Structurally plausible models for the fusion human interleukin-21 (IL-21) into the V1V2 loop of gp120 within a trimeric spike were essentially generated as described previously [42]. The trimeric configuration of gp120 in an unliganded spike was obtained by fitting the b12-bound conformation of HxBC2 core gp120 (PDB ID: 2NY7; [70]) into the cryoelectron tomography density of unliganded HIV-1 BaL virus using Chimera [71], [82]. RosettaDesign [83], [84] was used to thread the sequence of JRFL SOSIP.R6-IZ-H8 core gp120 onto the 2NY7 gp120 structure from residue 83 to 492. The structure of hIL-21 [63] was inserted into the V1V2 loop of the threaded model of JRFL SOSIP.R6-IZ-H8 core gp120 between residues 127 and 195 (gp120 2NY7 numbering), flanked by Gly-Ser-Gly (GSG) linkers on both sides. Domain insertion was carried out in the context of the gp120 trimer and in the presence of the b12 Fab bound to each of the three gp120 monomers to provide functional steric constraints during the modeling procedure.

### Env Trimer ELISA

Env trimer ELISA was performed as previously described [11], [66]. Briefly, supernatants containing His-tagged Env gp140 proteins were diluted 1∶3 in TBS (10 mM Tris, 150 mM NaCl, pH 7.5) supplemented with 10% FCS and added for 2 h to pre-blocked Ni-NTA HisSorb 96-well plates (Qiagen, Venlo, The Netherlands). After three washes using TSM (20 mM Tris, 150 mM NaCl, 1 mM CaCl_2_, and 2 mM MgCl_2_), serially diluted polyclonal HIV-Ig, Env specific monoclonal Abs, or CD4-IgG2, in TSM 5% BSA was then added for 2 h, with or without 1 µg/ml sCD4, followed by three washes with TSM, 0.05% Tween20. Horseradish peroxidase (HRP)-labeled goat-anti-human immunoglobulin G (0.2 µg/ml, Jackson Immunoresearch, Suffolk, UK) was used as secondary Ab and the absorption at 450 nm was measured after the colorimetric reaction was stopped using H_2_SO_4_. Equal input levels were verified SDS-PAGE followed by western blot analysis.

### Ig Secretion by Human B Cells

Human B cells were isolated from buffy coats of healthy donors obtained from the New York Blood Center by negative selection with B cell isolation kit II (Miltenyi Biotech, Auburn, USA). The purity of the sorted B cells was more than 97%, as assessed by CD19 staining. Purified B cells (5×10^4^) were plated in a 96-well U-bottom plate in 200 µl of complete RPMI 1640 medium containing 10% FBS, 2mM glutamine, 100U/ml streptomycin, 100U/ml penicillin, 1mM sodium pyruvate, and 10mM HEPES (all from Invitrogen, Carlsbad, USA). The cells were treated with 20 µl of 293T cell supernatant transfected with Env_wt_ or Env-fusion proteins in the presence of recombinant CD40L (Enzo Life Sciences, NY, USA) (200 ng/ml), interleukin-4 (IL-4) (R&D Systems, MN, USA) (10 ng/ml), and IL-10 (R&D Systems) (200 ng/ml) for 14 days. The amount of Env or Env-fusion proteins was normalized based on an anti-Env ELISA using the 2G12 MAb (data not shown) and adjusted using mock transfection supernatant. Recombinant human IL-21 (rhIL-21) was used at 10 ng/ml concentration. Culture supernatants were collected for the analysis of immunoglobulin secretion by an enzyme-linked immunosorbent assay (ELISA) (Bethyl Laboratories, Cambridge, UK). For fold-change calculations, the background levels of IgG, IgA and IgM secretion induced by the stimulation cocktail without Env or fusion proteins were subtracted from the test values and the fold-change was calculated compared to Env_wt_. Note that B cells cultured with supernatant of cleaved Env_IL-21_ co-transfected with a plasmid encoding furin were not viable; suggesting that residual furin in the supernatant had a toxic effect on these cells (data not shown). For this reason, the B cell experiments with cleavable Env and Env_IL-21_ were performed with proteins prepared without furin co-transfection.

### Flow Cytometry

B cells were immunophenotyped using fluorochrome-labeled MAb against CD38 (clone HIT2) and CD27 (clone M-T271) after 7 days of culturing with Env containing supernatant. The B cells were transferred into 96-well U-bottomed plates prior to staining and washed with ice-cold FACS buffer (PBS containing 2% heat-inactivated FBS) with centrifugation at 300×g at 4°C. B cells were stained for each MAb combination. MAb cocktails (50 µl) containing pre-titrated antibodies were added to B cells for 20 min on ice. Isotype-matched control MAbs were included in every assay. After staining, the B cells were washed twice and fixed in 1% paraformaldehyde. Two-color analysis was performed using a LSR II flow cytometer (BD Biosciences) and the results were analyzed in FlowJo software, version 9.4.3 (Tree Star, Ashland, OR, USA).

## Supporting Information

Figure S1 **Schematics and expression of the Env_IL-21_ and Env_ChimIL-21/4_.** Linear (A) and cartoon (B) representation of the Env_IL-21_ and Env_ChimIL-21/4_ constructs. HIV-1 Env molecule which has a chimeric IL-21/4 (ChimIL-21/4) cytokine molecule inserted in V1V2 domain (Env_ChimIL-21/4_) was designed by replacing amino acids 76 to 93 of IL-21, around helix C and the CD loop, with the homologous region of IL-4 (amino acids 87 to 98 of IL-4). (C) Env_IL-21_ and Env_ChimIL-21/4_ proteins expressed transiently in 293T cells were analyzed by reducing SDS-PAGE analysis followed by western blot.
(TIFF)

Figure S2 **Antigenic characterization of Env_IL-21_ and Env_ChimIL-21/4_.** ELISA reactivity of Env_IL-21_ and Env_ChimIL-21/4_ with 2G12 and HIV-Ig (A); b12 and CD4-IgG2 (B); and 48d (CD4i) in the absence and presence of sCD4 (C). All ELISA results are representative of at least three independent experiments using proteins derived from three independent transfections.
(TIFF)

Figure S3 **Immunoglobulin secretion from B cells cultured with Env_wt_, Env_IL-21_ and Env_ChimIL-21/4_ molecules supplemented with (A) CD40L/IL-10 and (B) CD40L/IL-4/IL-10.** Env_ChimIL-21/4_ augmented the secretion of IgG (4.4±2 & 2.4±0.8 µg/ml), IgA (1.78±0.5 & 0.66±0.13 µg/ml) and IgM (1.2±0.2 & 1±0.2 µg/ml) by human B cells. Culture supernatant from mock transfected 293T cells was used as a negative control. Data are representative of three independent experiments using B cells from three donors, each tested in duplicate.
(TIFF)

Figure S4 **Schematics (A) and expression (B) of Env_IL-21_ variants amino acid substitutions that modulate the interaction with the IL-21Rα and γC chains.** Immunoglobulin secretion from B cells cultured with Env_wt_, Env_IL-21_, and Env_IL-21_ variants in the presence of (C) CD40L/IL-10 and (D) CD40L/IL-4/IL-10. Data are representative of three experiments using B cells from different donors.
(TIFF)

Results S1 **Supporting results.**
(DOC)
