# Supplementary figures and images for: An HIV-1 Envelope Glycoprotein Trimer with an Embedded IL-21 Domain Activates Human B Cells
Source: PLoS One. 2013 Jun 24;8(6):e67309. doi: 10.1371/journal.pone.0067309 (PMC3691133; doi:10.1371/journal.pone.0067309)

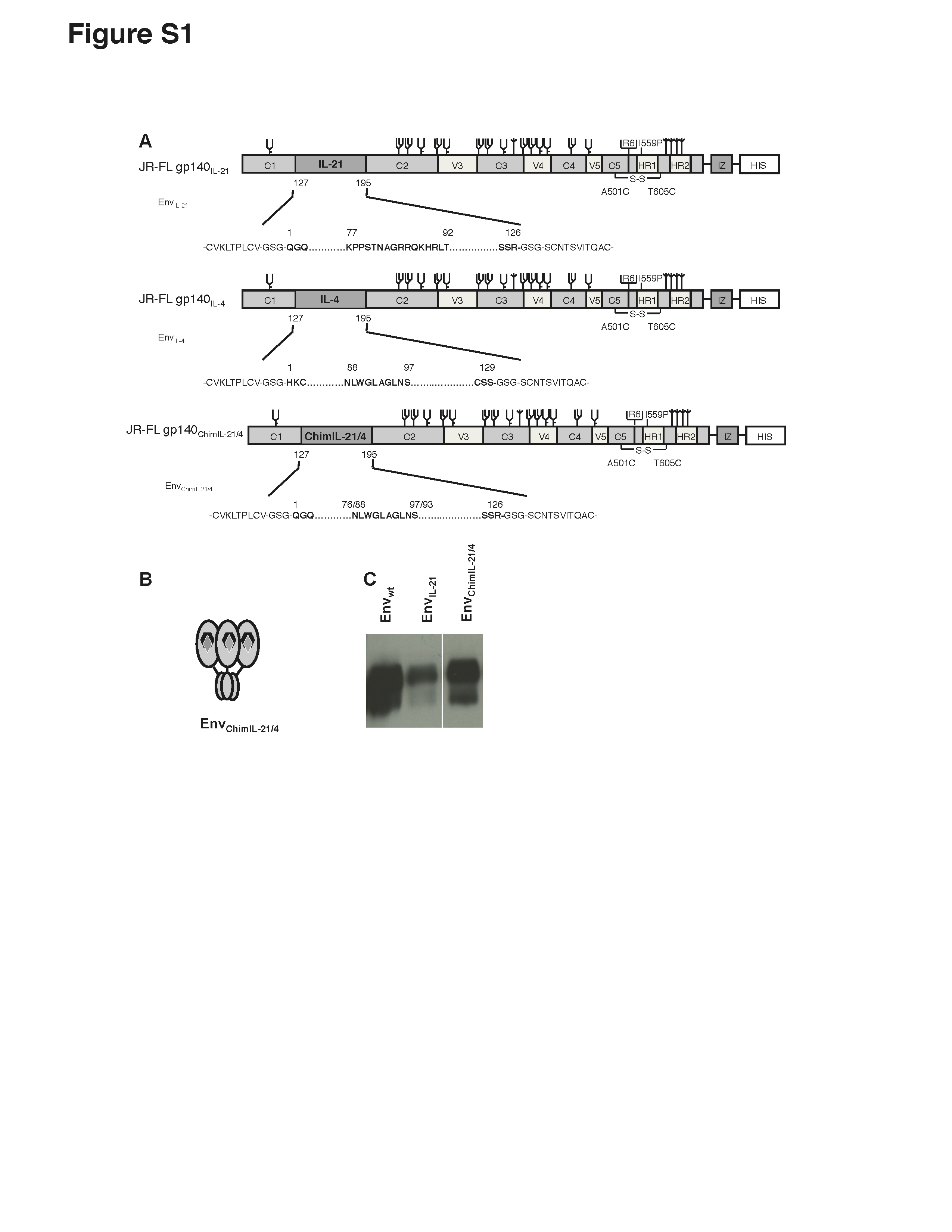

Supplement: Figure S1 — Schematics and expression of the EnvIL-21 and EnvChimIL-21/4. Linear (A) and cartoon (B) representation of the EnvIL-21 and EnvChimIL-21/4 constructs. HIV-1 Env molecule which has a chimeric IL-21/4 (ChimIL-21/4) cytokine molecule inserted in V1V2 domain (EnvChimIL-21/4) was designed by replacing amino acids 76 to 93 of IL-21, around helix C and the CD loop, with the homologous region of IL-4 (amino acids 87 to 98 of IL-4). (C) EnvIL-21 and EnvChimIL-21/4 proteins expressed transiently in 293T cells were analyzed by reducing SDS-PAGE analysis followed by western blot. (TIFF) [file pone.0067309.s001.tiff]

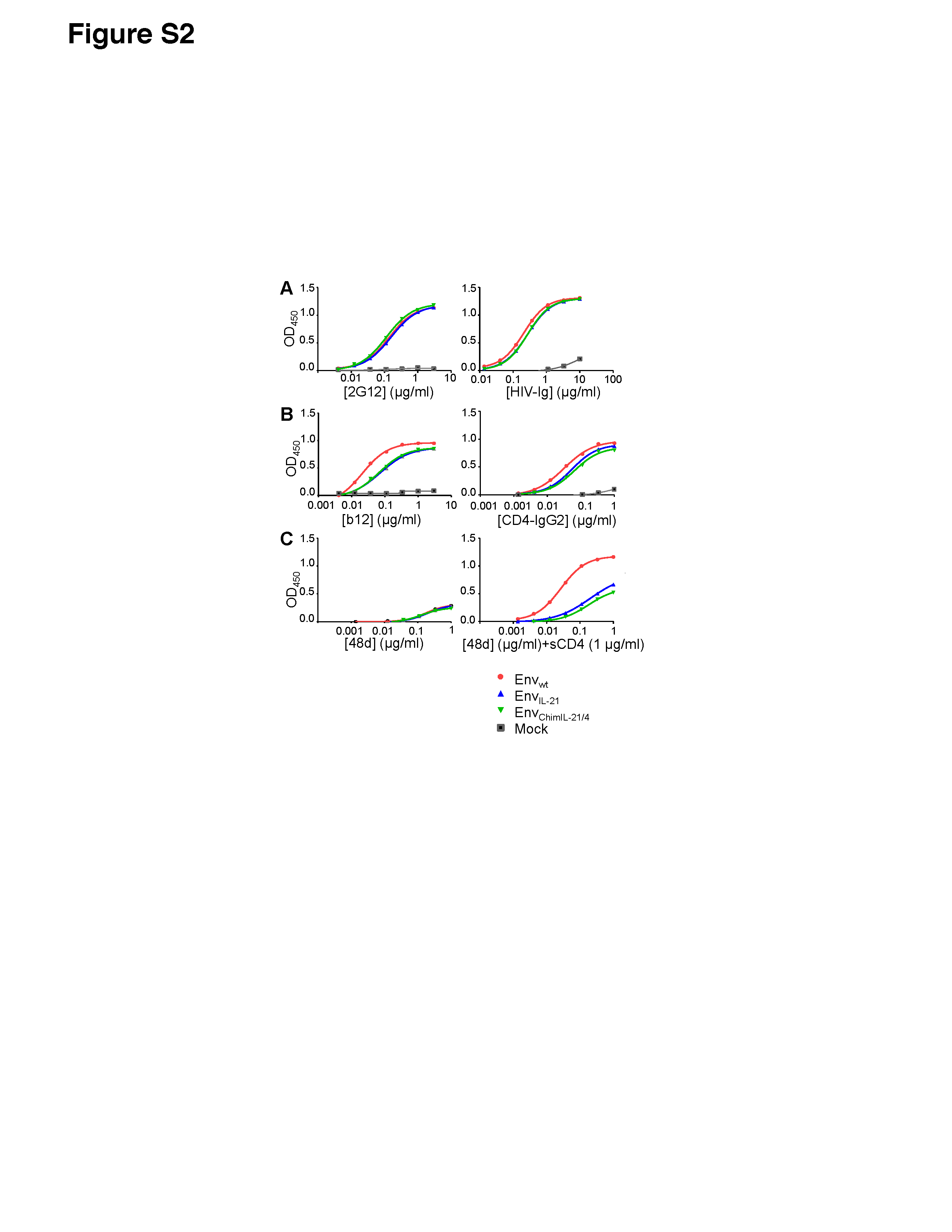

Supplement: Figure S2 — Antigenic characterization of EnvIL-21 and EnvChimIL-21/4. ELISA reactivity of EnvIL-21 and EnvChimIL-21/4 with 2G12 and HIV-Ig (A); b12 and CD4-IgG2 (B); and 48d (CD4i) in the absence and presence of sCD4 (C). All ELISA results are representative of at least three independent experiments using proteins derived from three independent transfections. (TIFF) [file pone.0067309.s002.tiff]

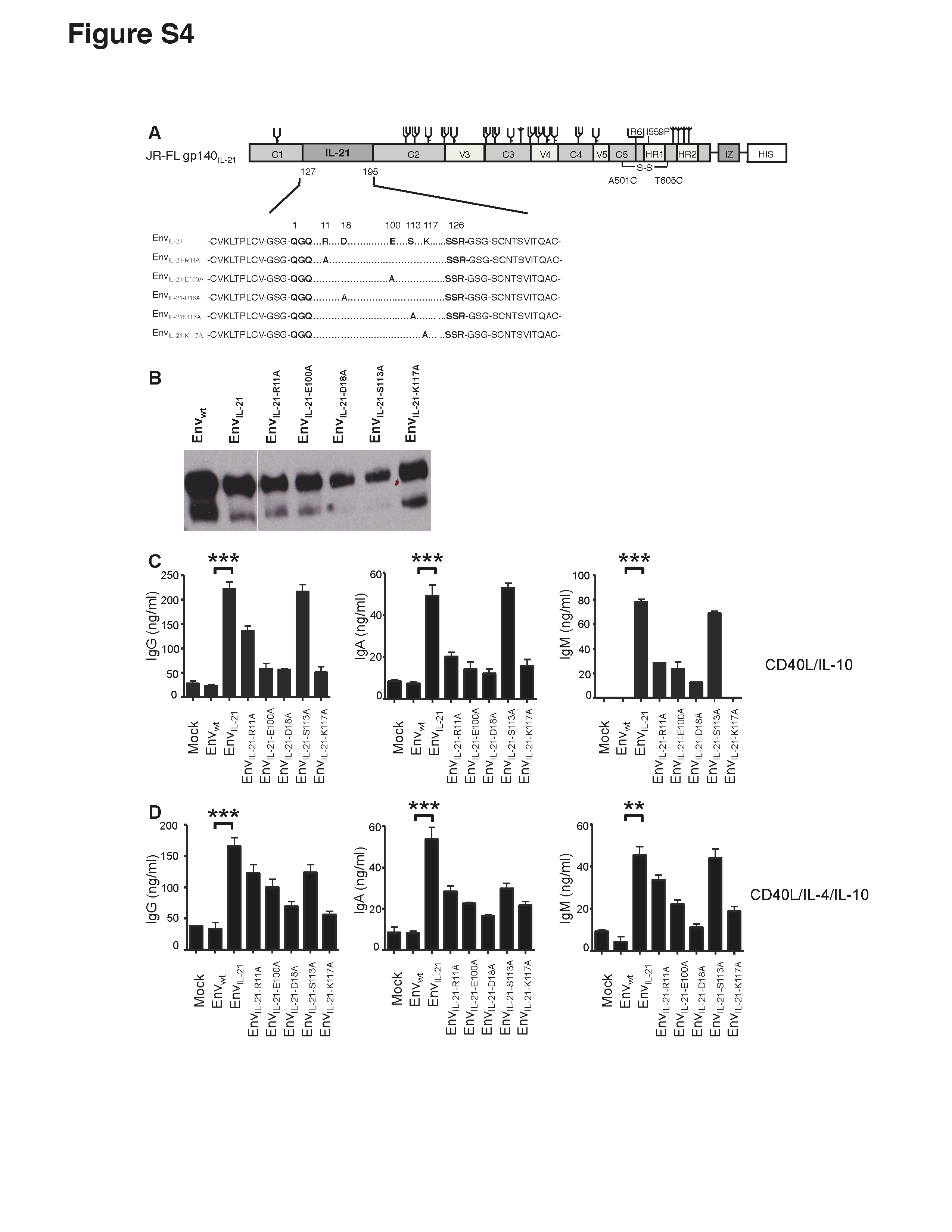

Supplement: Figure S4 — Schematics (A) and expression (B) of EnvIL-21 variants amino acid substitutions that modulate the interaction with the IL-21Rα and γC chains. Immunoglobulin secretion from B cells cultured with Envwt, EnvIL-21, and EnvIL-21 variants in the presence of (C) CD40L/IL-10 and (D) CD40L/IL-4/IL-10. Data are representative of three experiments using B cells from different donors. (TIFF) [file pone.0067309.s004.tiff]
