# Supplementary material for: An HIV-1 Envelope Glycoprotein Trimer with an Embedded IL-21 Domain Activates Human B Cells
Source: PLoS One. 2013 Jun 24;8(6):e67309. doi: 10.1371/journal.pone.0067309 (PMC3691133; doi:10.1371/journal.pone.0067309)
Supplement: Results S1 — Supporting results. (DOC) [file pone.0067309.s005.doc]

**Supplementary Results**

**PONE-D-13-14135**

**An HIV-1 envelope glycoprotein trimer with an embedded IL-21 domain activates human B cells**

Gözde Isik, Nancy P.Y. Chung, Thijs van Montfort, Sergey Menis, Katie Matthews, William R. Schief, John P. Moore, and Rogier W. Sanders

***Stabilizing helix C and the CD loop of IL-21 improves the expression but not the function of Env_IL-21_***

Replacing a structurally unstable region of IL-21 around helix C and the CD loop with the homologous and structurally more stable region from IL-4 can improve IL-21 signaling [1]. Based on this study, we designed an HIV-1 Env molecule which has a chimeric IL-21/4 (ChimIL-21/4) cytokine molecule inserted in V1V2 domain (Env_ChimIL-21/4_)(Fig. S1A & S1B). Env_ChimIL-21/4_ was expressed as efficiently as Env_wt_, but notably better than the original Env_IL-21_ molecule (Fig. S1C). The antigenic structure of Env_ChimIL-21/4_ molecule was similar to Env_IL-21_ (Fig. S2B & S2C). However, we did not observe a consistent enhancement in immunoglobulin secretion compared to Env_IL-21_ (Fig. S3A & S3B). We also assessed the induction of plasmablast-like cells by measuring the induction of CD38 and CD27 cultured with Env_ChimIL-21/4_ (Fig. 3E). Although Env_ChimIL-21/4_ efficiently induced the upregulation of both markers, there was not significant increase in plasmablast-like cells in Env_ChimIL-21/4_ (25% double positive cells) compared to Env_IL-21_ (26%), but there was a significant increase in CD38 expression (Fig. 3F). Thus, although Env_ChimIL-21/4_ was expressed more efficiently than Env_IL-21_, which may be related to the local domain stabilization around helix C and the CD loop, it was not more potent than Env_IL-21_ in activating human B cells.

***Modulating the interaction with the IL-21 receptor decreases Env_IL-21_ activity***

In a further attempt to increase the activity of the IL-21 domain of Env_IL-21_, we introduced several substitutions at IL-21 residues that are critical for receptor interactions and that have been shown to increase the affinity for either the IL-21Rα or γC chain (Fig. S4A) [2]. We selected R11A and E100A, which have higher affinity for the IL-21Rα chain, and D18A, S113A and K117A, which have higher affinity to the γC chains [2]. The single amino acid mutant Env_IL-21_ molecules expressed similarly or slightly less efficiently than the original Env_IL-21_, and all of the mutants were expressed less efficiently than Env_wt_ (Fig. S4B). We tested the effect of these substitutions on the activation of primary human B cells. The S113A mutant was as potent as the unmodified Env_IL-21_, at inducing immunoglobulin secretion from B cells, but the other modified molecules were all less potent (Fig. S4C & S4D).

Reference List

1. Bondensgaard K, Breinholt J, Madsen D, Omkvist DH, Kang L, Worsaae A, Becker P, Schiodt CB, Hjorth SA (2007) The existence of multiple conformers of interleukin-21 directs engineering of a superpotent analogue. J Biol Chem 282: 23326-23336. M701313200 [pii];10.1074/jbc.M701313200 [doi].

2. Kang L, Bondensgaard K, Li T, Hartmann R, Hjorth SA (2010) Rational design of interleukin-21 antagonist through selective elimination of the gammaC binding epitope. J Biol Chem 285: 12223-12231. M110.101444 [pii];10.1074/jbc.M110.101444 [doi].
